# A novel method for predicting hepatocellular carcinoma response to chemoembolization using an intraprocedural CT hepatic arteriography-based enhancement mapping: a proof-of-concept analysis

**DOI:** 10.1186/s41747-022-00315-8

**Published:** 2023-01-30

**Authors:** Ryosuke Taiji, Yuan-Mao Lin, Gouthami Chintalapani, Ethan Y. Lin, Steven Y. Huang, Armeen Mahvash, Rony Avritscher, Chien-An Liu, Rheun-Chuan Lee, Vivian Resende, Hideyuki Nishiofuku, Toshihiro Tanaka, Kimihiko Kichikawa, Ernst Klotz, Sanjay Gupta, Bruno C. Odisio

**Affiliations:** 1 Division of Diagnostic Imaging, Department of Interventional Radiology, The University of Texas MD Anderson Cancer Center, 1515 Holcombe Blvd, Houston, TX 77030 USA; 2 Department of Radiology and Nuclear Medicine, Nara Medical University, Kashihara, Nara, 634-8521 Japan; 3 Siemens Medical Solutions USA Inc, 501 North Barrington Road, Hoffman Estates, IL 60192 USA; 4 Department of Radiology, Taipei Veterans General Hospital, Taipei, 112 Taiwan; 5 College of Medicine, National Yang Ming Chiao Tung University, Taipei, Taiwan; 6 Department of Surgery, Federal University of Minas Gerais, Belo Horizonte, MG Brazil; 7 Siemens Healthineers, Siemensstraße 3, 91301 Forchheim, Germany

**Keywords:** Carcinoma (hepatocellular), Chemoembolization (therapeutic), Computed tomography angiography, Cone-beam computed tomography, Microspheres

## Abstract

**Background:**

To evaluate the feasibility of a novel approach for predicting hepatocellular carcinoma (HCC) response to drug-eluting beads transarterial chemoembolization (DEB-TACE) using computed tomography hepatic arteriography enhancement mapping (CTHA-EM) method.

**Methods:**

This three-institution retrospective study included 29 patients with 46 HCCs treated with DEB-TACE between 2017 and 2020. Pre- and posttreatment CTHA-EM images were generated using a prototype deformable registration and subtraction software. Relative tumor enhancement (*T*_Post/pre-RE_) defined as the ratio of tumor enhancement to normal liver tissue was calculated to categorize tumor response as residual (*T*_Post-RE_ > 1) *versus* non-residual (*T*_Post-RE_ ≤ 1) enhancement, which was blinded compared to the response assessment on first follow-up imaging using modified RECIST criteria. Additionally, for tumors with residual enhancement, CTHA-EM was evaluated to identify its potential feeding arteries.

**Results:**

CTHA-EM showed residual enhancement in 18/46 (39.1%) and non-residual enhancement in 28/46 (60.9%) HCCs, with significant differences on *T*_Post-RE_ (3.05 ± 2.4 *versus* 0.48 ± 0.23, respectively; *p* < 0.001). The first follow-up imaging showed non-complete response (partial response or stable disease) in 19/46 (41.3%) and complete response in 27/46 (58.7%) HCCs. CTHA-EM had a response prediction sensitivity of 94.7% (95% CI, 74.0–99.9) and specificity of 100% (95% CI, 87.2–100). Feeding arteries to the residual enhancement areas were demonstrated in all 18 HCCs (20 arteries where DEB-TACE was delivered, 2 newly developed collaterals following DEB-TACE).

**Conclusion:**

CTHA-EM method was highly accurate in predicting initial HCC response to DEB-TACE and identifying feeding arteries to the areas of residual arterial enhancement.

**Supplementary Information:**

The online version contains supplementary material available at 10.1186/s41747-022-00315-8.

## Keypoints

- Computed tomography hepatic arteriography enhancement mapping (CTHA-EM) correlated with initial hepatocellular carcinoma response to drug-eluting beads transarterial chemoembolization (DEB-TACE).
- CTHA-EM provided quantitative evaluation of residual arterial tumor enhancement after DEB-TACE.
- CTHA-EM identified feeding arteries to areas of residual tumor enhancement.
- CTHA-EM might be used to guide treatment delivery and determine treatment endpoint.

## Background

Transarterial chemoembolization (TACE) is a standard treatment for patients with intermediate-stage hepatocellular carcinoma (HCC) [1, 2]. Its oncological effects are related to tumor arterial devascularization and necrosis, which do not prompt immediate changes in overall tumor size. Consequently, arterial enhancement-based treatment response criteria are considered to be the reference standard for TACE response evaluation [3–5] and have demonstrated superiority over size-based criteria for predicting pathological response and survival [4, 6–8].

In lipiodol-based TACE, the degree of intratumoral lipiodol accumulation can be utilized as a surrogate for treatment response and can be assessed by intraprocedural computed tomography (CT) or cone-beam CT (CBCT) [9–12]. In drug-eluting beads TACE (DEB-TACE), tumor response can be estimated to a certain extent by the degree of contrast agent accumulated within the tumor during the delivery of DEB-TACE on native CT or CBCT acquired immediately after treatment [13]. Nevertheless, as opposed to lipiodol, such contrast agent retention occurs only transiently and may not be a reliable surrogate for assessing embolization endpoint and predicting treatment response. Furthermore, in the event of acquiring a contrast-enhanced CT/CBCT arteriography after DEB-TACE, contrast agent retained in the tumor might obscure areas of residual tumor enhancement making it hard to identify true residual tumor enhancement. The use of dual-phase CBCT with perfusion blood volume imaging for response prediction has been reported for patients undergoing DEB-TACE [14–16]. However, its use in clinical practice is limited due to the lack of a reliable and reproducible method to assess residual tumor enhancement, often attributed to the complexity of acquiring CBCT images, lack of standardization of gray scale values, and the sensitivity of CBCT acquisition to motion and breathing artifacts. Finally, none of those reported methods identifies the putative artery responsible for residual tumor enhancement, limiting its application for intra-procedure decision-making. Therefore, two-dimensional digital subtraction angiography (DSA) remains to be the primary method of subjectively assessing DEB-TACE treatment endpoint.

Advanced algorithms that apply deep learning methods on DSA image sequence are being developed to predict treatment response [17]. However, DSA imaging due to its inherent projection geometry poses many challenges in identifying residual tumor enhancement depending on the amount and location of the residual tumor, number of hepatic arteries supplying it, subtraction artifacts from respiratory and cardiac motion, and end point assessment has been reported to be highly variable between operators [18–20]. Several studies have demonstrated the importance of achieving adequate embolization during TACE and its correlation to improved survival outcomes, with emphasis on achieving complete response at first TACE session [18, 21, 22] and CT-based texture analysis predictive modeling to select optimal patients for TACE intervention upfront [23]. Hence, a robust, accurate, and objective method of predicting early treatment response to TACE remains the most desirable need.

CT hepatic arteriography (CTHA) has proved to be superior to CBCT for TACE planning because of its better contrast resolution, comparable spatial resolution, and minimal artifacts caused by motion and beam hardening [24]. CTHA also permits reliable quantification of parenchymal enhancement in Hounsfield units (HU), potentially allowing an objective and reproducible method for assessing TACE treatment endpoint. Several angiography/CT users have been using repeated CTHA imaging to identify tumor feeding arteries as well as residual tumor areas [25]. However, it is often difficult to delineate contrast stasis and residual tumor.

The aim of this study was to evaluate the feasibility of a novel approach to predict HCC response to DEB-TACE using CTHA enhancement mapping (CTHA-EM) through image subtraction.

## Methods

### Study population

**Fig. 1:**
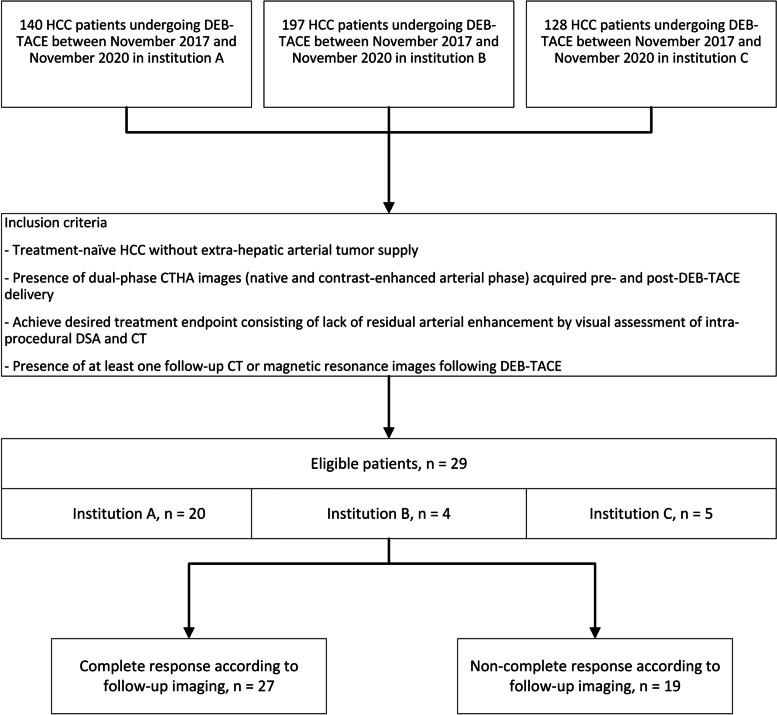
Participant flowchart for inclusion

This three-institution retrospective study was compliant with the Health Insurance Portability and Accountability Act and approved by each institutional review board with a waiver of informed consent. Between November 2017 and November 2020, the prospectively compiled DEB-TACE registries were searched to identify patients who met the following inclusion criteria: (1) treatment-naïve HCC without extrahepatic arterial tumor supply, (2) who were treated with DEB-TACE and had a dual-phase CTHA images (native and contrast-enhanced arterial phases) acquired before and after DEB-TACE delivery (*n* = 33 patients), (3) who were deemed to have achieved complete response (CR) by intra-procedural DSA and CT (*n* = 30 patients), and (4) who had at least one follow-up CT or magnetic resonance imaging following DEB-TACE (Fig. 1).

### DEB-TACE protocol

Six interventional radiologists (B.C.O., A.M., S.T., T.M., H.N., and C.A.L.), with 9, 15, 8, 9, 18, and 11 years of experience, respectively, performed DEB-TACE in an angio/CT suite. After conscious sedation, transarterial access was obtained with femoral artery puncture, and the target tumor(s) and respective feeding hepatic arteries were identified by DSA and CTHA. The feeding arteries were selected on a segmental or subsegmental level with a 1.1–2.4-Fr microcatheter (Progreat, Terumo, Japan; PIXIE, Tokai, Japan, or Parkway, Asahi, Japan). DEB-TACE was performed with 70−150 or 100−300 μm particles (LC Bead *M1*, Boston Scientific, USA, or DC Beads, Eisai, Japan) loaded with doxorubicin (25 mg/mL, 50 mg total) or epirubicin (50 mg/mL, 75 mg total) and mixed with 12, 15, or 19 mL of nonionic contrast medium and 6, 5, or 10 mL of 0.9% saline, respectively. The solution was injected manually at a rate of approximately 1 mL/min until complete tumor devascularization and near stasis of the feeding artery was documented on DSA images. All DEB-TACE were performed with the goal of achieving CR per modified response evaluation criteria in solid tumors (mRECIST) [4].

### Intraprocedural CTHA imaging protocol

A dual-phase CTHA (native CT and intra-arterial CT during hepatic arteriography) was routinely acquired for DEB-TACE planning to evaluate the presence of additional tumors, identify target tumor(s) and their feeding arteries, and rule out the presence of extrahepatic feeding arteries. CTHA was performed using contrast agent (Omnipaque 300, General Electric Healthcare, Chalfont, St. Gille, UK) injected at 2 mL/s (average total volume 22 mL) with an acquisition delay of 4 or 8 s for arterial phase using a 5-Fr catheter placed in the celiac artery, common hepatic artery, or left gastric artery, or a 1.1−2.4-Fr coaxial microcatheter placed in the common hepatic artery or proper hepatic artery (Supplementary Table 1). Immediate CTHA after treatment was acquired at the discretion of the interventional radiologists to complement subjective assessment of the treatment using DSA images in cases where DSA images were not adequate to determine the treatment endpoint.

### CTHA-EM imaging processing

**Fig. 2:**
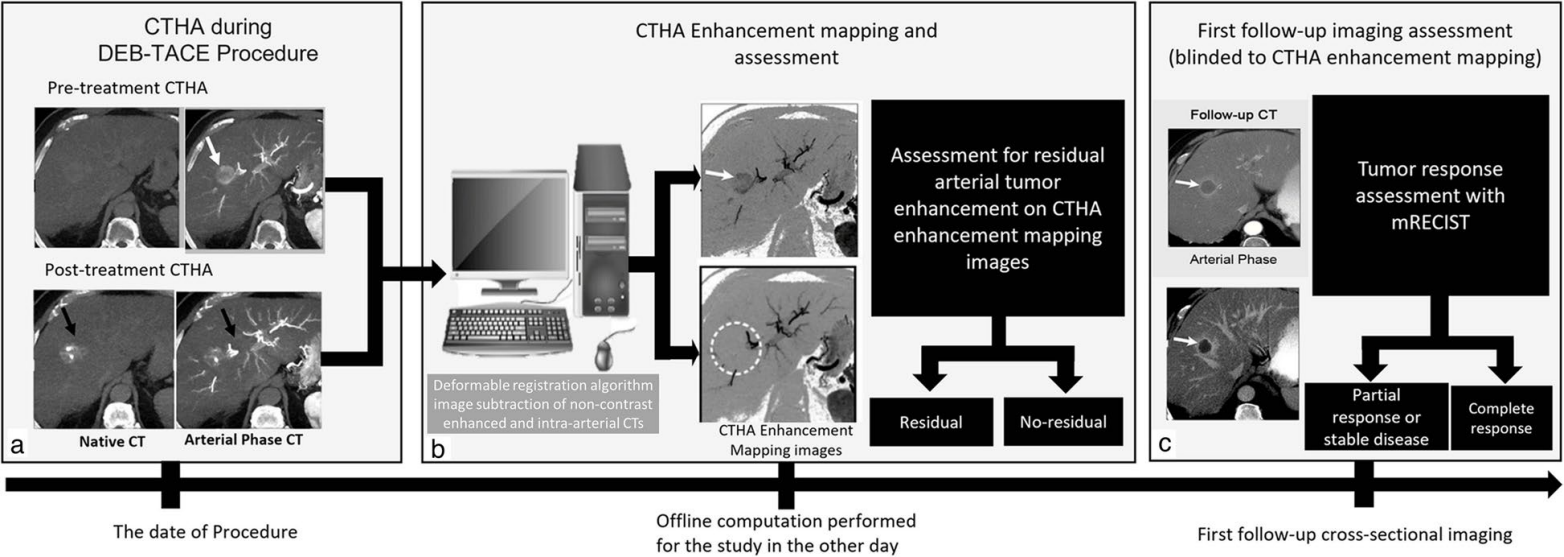
Schematic illustration of proposed CTHA-EM method to predict hepatocellular carcinoma response to DEB-TACE. **a** CTHA image acquisition during DEB-TACE consists of dual-phase CT (native- and contrast-enhanced arterial phases) acquired before and after DEB-TACE. Pre-treatment CTHA showed hypervascular tumor in segment 8 (white arrow). Posttreatment CTHA showed contrast agent accumulation within the tumor from DEB-TACE along with arterial supply (black arrow), making it difficult to delineate contrast stasis from residual tumor blush. **b** Pre- and posttreatment dual-phase CTHA images were loaded into a workstation, and software was used to generate CTHA-EM images with deformable registration and subtraction to assess residual tumor enhancement. The tumor was highlighted on pre-treatment CTHA enhancement mapping (white arrow), and no residual tumor enhancement was depicted on the posttreatment CTHA enhancement mapping (dotted circle). Contrast stasis from the posttreatment native CT was subtracted from the arterial phase to show potential residual tumor arterial enhancement. **c** First follow-up computed tomography imaging after DEB-TACE was used to predict the treatment response accordingly to mRECIST, confirming complete response in this case. *CTHA*, Computed tomography hepatic arteriography; *CTHA-EM* CTHA, enhancement mapping; *DEB-TACE*, Drug-eluting beads transarterial chemoembolization; *mRECIST*, Modified Response Evaluation Criteria in Solid Tumors

A CTHA-EM algorithm was applied to pre- and posttreatment CTHA images with an offline prototype software (Hepacare, Siemens Healthineers, Germany). CTHA-EM analysis was not performed at the time of the procedure and was not used for intraprocedural decision-making. First, automatic registration using a deformable registration algorithm (Fig. 2) [26–29] was performed to establish a voxel-level mapping between the non-contrast and arterial phase CTHA images. Registration accuracy assessment of this algorithm was reported to be 1.3 ± 1.1 mm on average, with larger errors (1.9 ± 1.7 mm) seen on the periphery of the liver. We did not assess the registration accuracy of the algorithm as it was beyond the scope of this paper, and our datasets were acquired back to back with exact imaging acquisition settings, less prone to artifacts from breathing/cardiac motion, and very minimal liver deformation. Second, registered images were subtracted to uncover true tumor enhancement. These two steps took less than 30 s and were performed on pre- and post-images separately to create pre- and posttreatment CTHA-EM images, respectively. Third, pre- and posttreatment CTHA-EM images were co-registered to facilitate voxel-based comparison and to segment HCC on the posttreatment CTHA-EM images. Processing was done on the full quasi-isotropic high-resolution data (voxel size, 0.6 mm^3^) without any smoothing to retain full vascular contrast.

### CTHA-EM analysis and interpretation

**Fig. 3:**
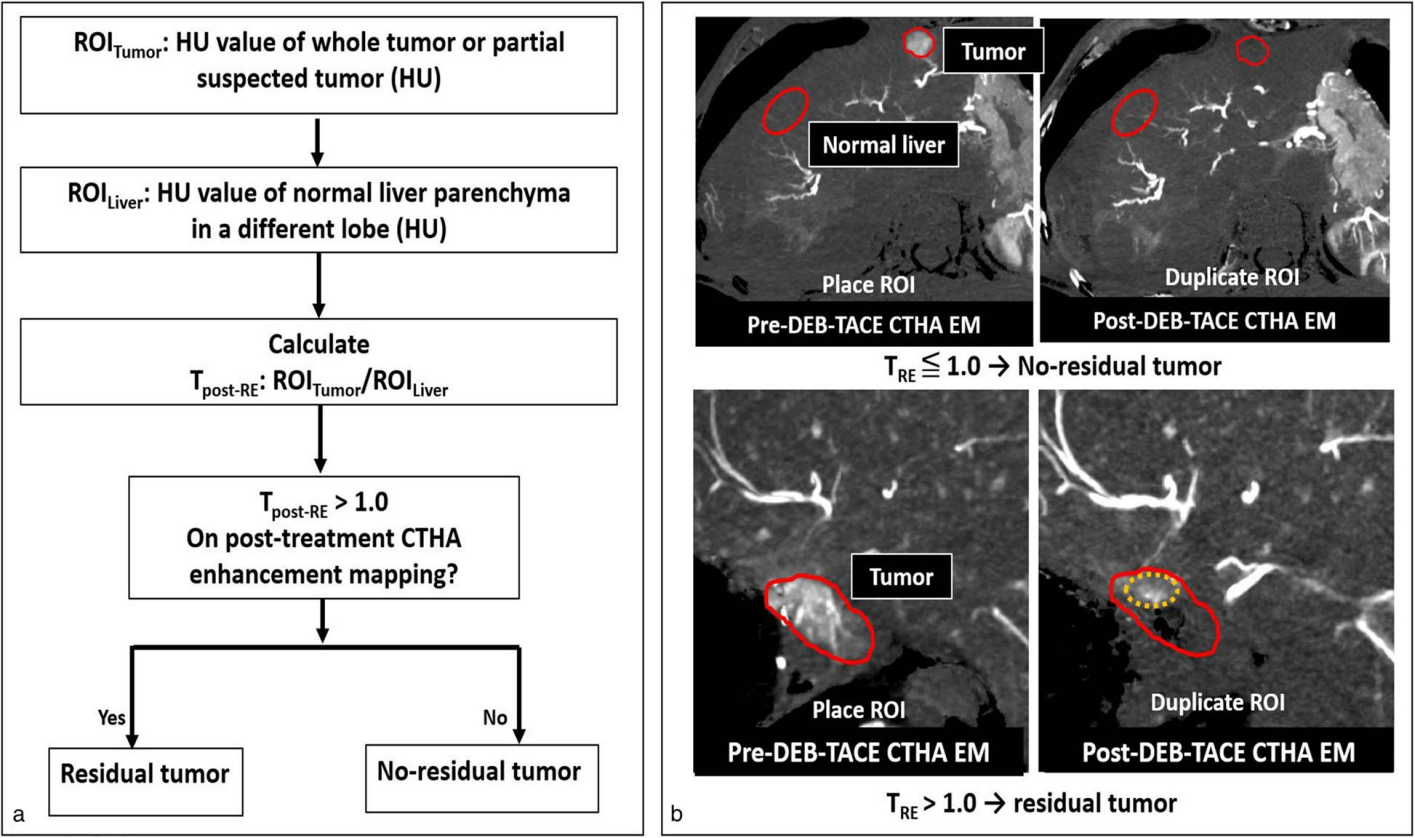
Flowchart and two examples illustrating the proposed CTHA-EM ROI-based quantitative analysis.** a** Flowchart of quantitative assessment of residual tumor enhancement using CTHA-EM. **b** Two cases exemplifying ROI-based quantitative analysis. Top row: *ROI*_Tumor_ (solid red circle) and *ROI*_normal_ (dotted red circle) were placed on pre-treatment CTHA-EM images and transferred to the corresponding anatomical location on posttreatment CTHA-EM images. Bottom row: For cases with suspected residual enhancement, voxels with higher attenuation (orange dotted circle) were used for *T*_Post-RE_ to avoid normalization bias from zero voxels due to subtraction of contrast stasis. *T*_post-RE_, Relative enhancement of tumor on posttreatment images; *CTHA-EM*, Computed tomography hepatic arteriography enhancement mapping; *ROI*, Region of interest

CTHA-EM analysis was conducted by a computer scientist (G.C.) and an imaging physicist (E.K.), independent of any interventional radiologists. Furthermore, such analysis was subsequently verified by an interventional radiologist board-certified in diagnostic and interventional radiology (B.C.O.) prior to the first follow-up imaging. Quantitative assessment was made by measuring the mean HU values in regions of interest (ROIs) on CTHA-EM images (Fig. 3). ROIs were drawn on pre-treatment CTHA-EM images (*ROI*_Tumor_ — largest tumor cross-sectional area as seen on either axial or coronal plane; *ROI*_Normal_ — normal liver parenchyma in the contralateral hepatic lobe, excluding blood vessels) and transferred to posttreatment CTHA-EM image. Tumor relative enhancement on pre-(*T*_Pre-RE_) and post-(*T*_Post-RE_) DEB-TACE CTHA-EM images was calculated as the ratio of HU values of tumor enhancement to the normal non-embolized liver parenchyma (*ROI*_Tumor_/*ROI*_Normal_). Tumor response to DEB-TACE was defined as no residual (*T*_Post-RE_ ≤ 1) *versus* residual tumor enhancement (*T*_Post-RE_ > 1).

CTHA-EM-imaging-based response prediction was interpreted and recorded blinded to the first follow-up imaging (FUI). Finally, for tumors with residual enhancement on posttreatment CTHA-EM images, maximum intensity projection (5−10 mm thin) images were used to identify the potential feeding arteries supplying the residual tumor enhancement areas, which was defined by the presence of an artery leading to the area of residual tumor enhancement. In addition, pre-treatment CTHA imaging was evaluated to identify if such feeding arteries were present or if they were new collaterals that developed following DEB-TACE delivery.

### Follow-up imaging and the treatment response

The first follow-up imaging after DEB-TACE was performed either with magnetic resonance imaging (MRI) or CT quadriphasic protocol according to the American Association for the Study of Liver Diseases guidelines [2]. Two interventional radiologists (E.Y.L. and S.Y.H.), with 8 and 9 years of experience, the later one being a board-certified in diagnostic and interventional radiology, blinded to CTHA-EM, assessed the per-tumor treatment response according to mRECIST criteria.

### Statistical analysis

The study primary outcome measure was to evaluate the ability of CTHA-EM in predicting the per-tumor treatment response at the first follow-up imaging. Secondary outcome was to correlate the exact anatomical areas of residual tumor on CTHA-EM with the first follow-up imaging and to explore the capability of CTHA-EM in identifying the feeding arteries to residual tumors. Quantitative evaluation of tumor relative enhancement pre- and post-DEB-TACE (HU and percentage change) between treatment response cohorts was performed using the Wilcoxon rank-sum test. To calculate specificities, sensitivities, positive predictive values (PPV), and negative predictive values (NPV) with 95% confidence intervals (CI), we used cross-tabulation. Values of *p* lower than 0.05 were considered statistically significant. Statistical analysis was performed by using commercially available statistical software (SPSS, v.24; IBM, Armonk, USA).

## Results

A total of 29 patients (24 men, mean age 68 years, range 50−87) with 46 HCCs (mean diameter 2.7 cm, range 0.6–6.3) submitted to twenty-nine DEB-TACE sessions met the inclusion criteria. Demographic and clinical characteristics of all the patients are shown in Table 1.

**Table 1:** Baseline demographics and clinical characteristics of 29 patients who underwent DEB-TACE

Characteristics	Value
Sex	
Male	24 (83)
Female	5 (17)
Age, mean ± SD (range), years	68 ± 8.0 (50–87)
Tumor size, median (IQR), cm	2.4 (1.4–3.5)
Number of tumors treated with DEB-TACE, per patient	
One	16 (55)
Two	9 (31)
Three	4 (14)
AST, median (IQR), IU/L	42 (26–58)
ALT, median (IQR), IU/L	34 (21–47)
Total bilirubin, median (IQR), mg/dL	0.8 (0.5–1.1)
Albumin, mean ± SD, g/dL	3.9 ± 0.5
INR, median (IQR)	1.1 (0.9–1.3)
Child-Pugh grade, per patient	
A	26 (90)
B	3 (10)
Catheter tip location during CTHA, per patient	
Celiac trunk	9 (31)
Common hepatic artery	17 (59)
Proper hepatic artery	1 (3)
Others	2 (7)
Interval between DEB-TACE session and first follow-up imaging, median (IQR), weeks	6.7 (3.7−9.7)
Modality of imaging follow-up	
CT	19 (66)
MRI	9 (31)
CT and MRI	1 (3)

Continuous data are expressed as mean ± standard deviation (SD) or median (IQR), categorical data as number of patients (percentage). *ALT* Alanine aminotransferase, *AST* Aspartate aminotransferase, *CTHA* Computed tomography during hepatic arteriography, *DEB-TACE* Drug-eluting beads transarterial chemoembolization, *INR* International normalized ratio, *IQR* Interquartile range, *MRI* Magnetic resonance imaging

Quantitative evaluation of tumor relative enhancement (*T*_Pre-RE_ and *T*_Post-RE_) showed significant differences between residual and no-residual enhancement groups after DEB-TACE (mean ± standard deviation 3.05 ± 2.4 *versus* 0.48 ± 0.23, respectively, *p* < 0.001). No significant differences in HU were observed in HCCs between no-residual and residual enhancement before treatment (mean ± standard deviation 3.66 ± 2.7 *versus* 3.68 ± 2.0, *p* = 0.671), indicating that the posttreatment CT attenuation values of the residual enhancement were similar to the pre-treatment values. Per-tumor response analysis based on CTHA-EM (*T*_Post-RE_) showed 18/46 (39.1%) HCCs that had residual enhancement, while 28/46 (60.9%) had no residual arterial enhancement.

**Fig. 4:**
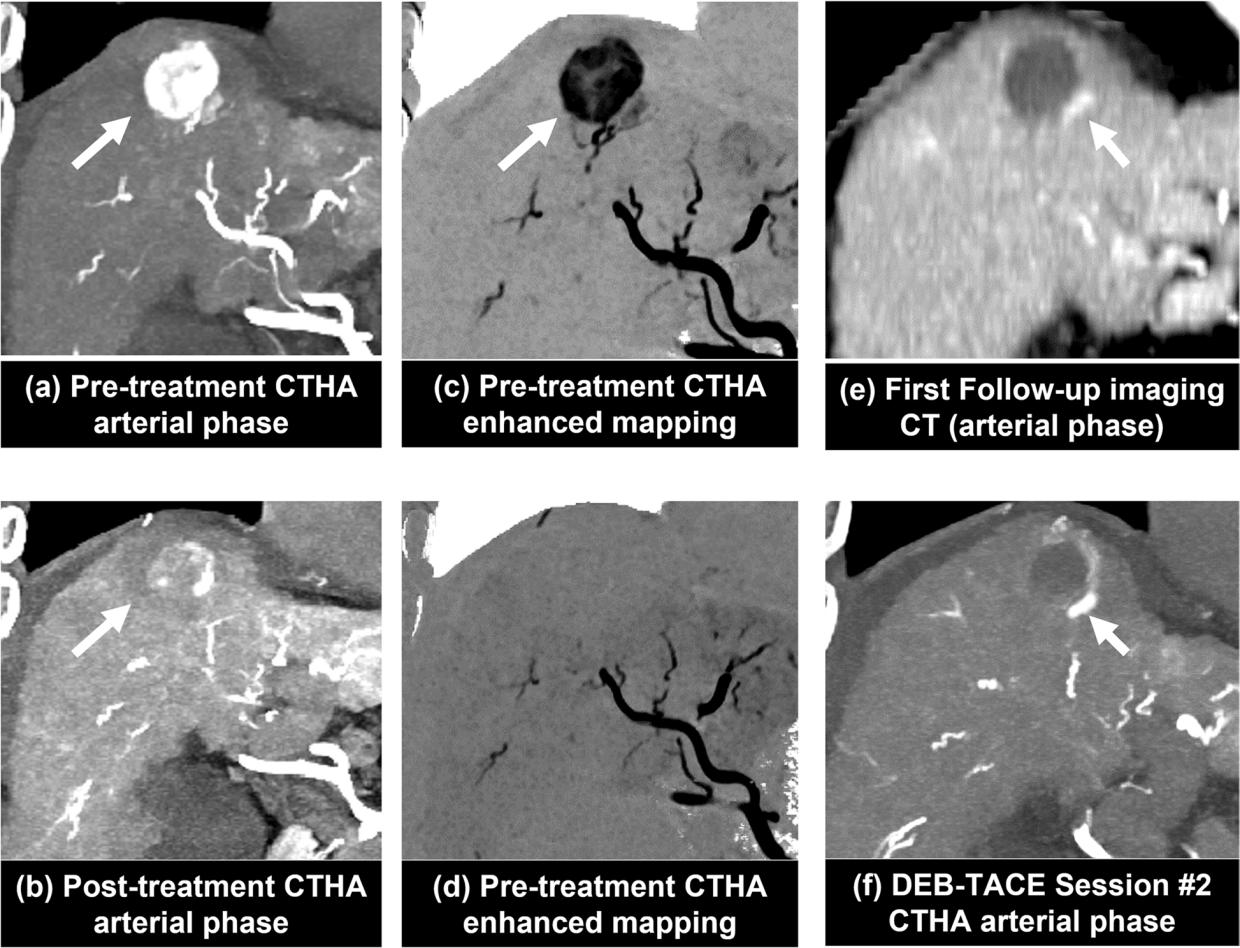
A false-negative case for no-residual enhancement on CTHA-EM analysis showing residual tumor on first follow-up imaging 13.3 weeks later. **a** Pretreatment CTHA arterial phase showed hypervascular HCC (white arrow). **b** Posttreatment CTHA showed retention of contrast media in the tumor (white arrow). **c** Pre-treatment CTHA-EM image shown in inverted gray scale with HCC (white arrow). **d** Posttreatment CTHA-EM demonstrated lack of residual tumor enhancement at the target HCC or its feeding artery, therefore identified as “no-residual.” **e** First follow-up CT imaging (arterial phase) showed residual tumor (white arrow). **f** Second session of pretreatment CTHA confirmed residual tumor along the border of treated tumor (white arrow). Tumor progression instead of residual untreated tumor cannot be excluded due to long follow-up period from DEB-TACE procedure to first follow-up imaging. *CTHA*, Computed tomography hepatic arteriography; *CTHA-EM*, CTHA enhancement mapping; *DEB-TACE*, Drug-eluting beads transarterial chemoembolization; *HCC*, Hepatocellular carcinoma

The median interval from DEB-TACE to the follow-up imaging was 6.7 weeks (interquartile range 3.7−9.7). The first follow-up imaging showed complete response in 27 (58.7%), partial response in 17 (37.0%), and stable disease in 2 (4.3%) of the 46 HCCs. Table 2 shows the correlation between CTHA-EM imaging and the first follow-up imaging tumor response assessment. Treatment response prediction by CTHA-EM images yielded a sensitivity of 94.7% (95% CI, 74.0–99.9), specificity of 100.0% (95% CI, 87.2−100), PPV of 100% (95% CI, 79.3–100), NPV of 96.4% (95% CI, 80.0–99.5), and an accuracy of 97.8% (95% CI, 88.5–99.9). Based on CTHA-EM analysis, there was only one false-negative case for residual tumor enhancement, identified as PR on the follow-up imaging, of all the 46 tumors (Fig. 4).

**Table 2:** Diagnostic accuracy of intra-procedural CTHA-EM

		First follow-up imaging, mRECIST	
		Non-complete response, *i.e.,* partial response or stable disease (*n* = 19)	Complete response (*n* = 27)
CTHA-EM	Residual tumor (*n* = 18)	18	0
	Non-residual tumor (*n* = 28)	1	27

*CTHA-EM* Computed tomography hepatic arteriography enhancement mapping, *mRECIST* Modified response evaluation criteria in solid tumors

**Fig. 5:**
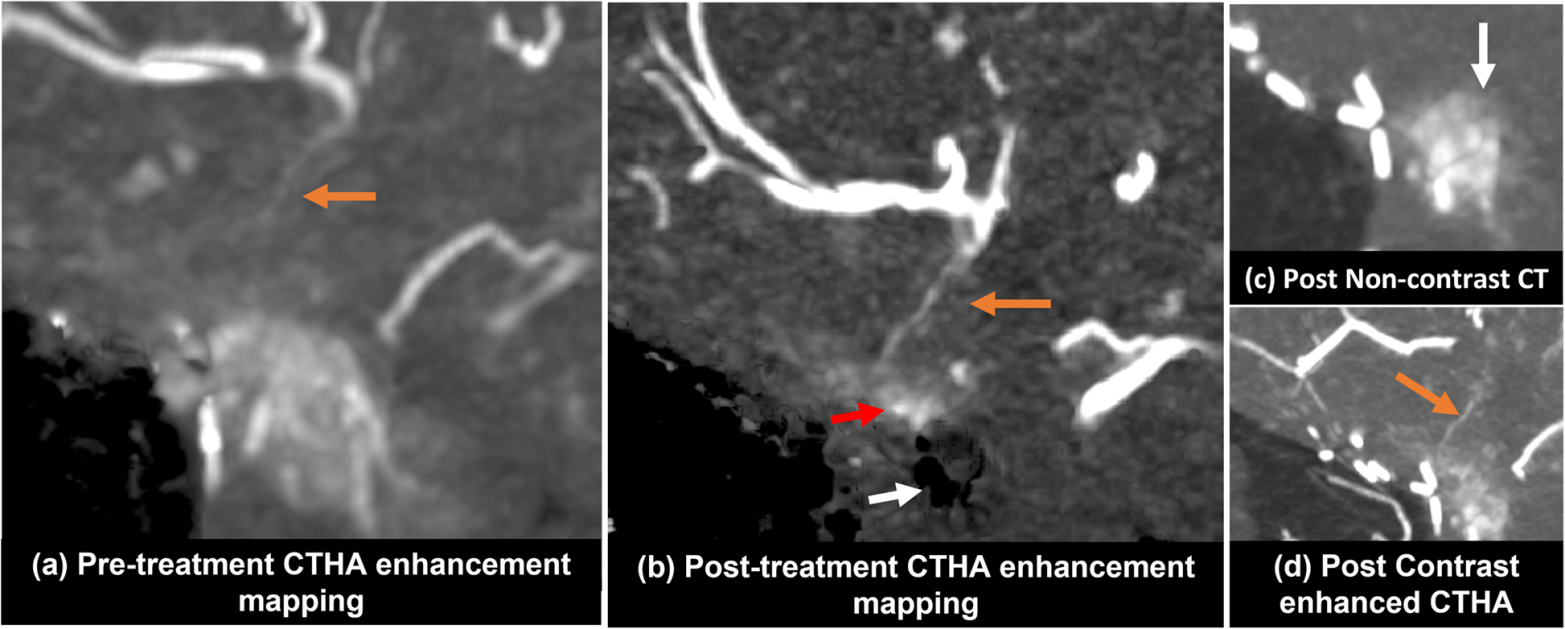
An example illustrating how to identify feeding arteries on CTHA-EM with residual tumor enhancement. **a** Before DEB-TACE, a small feeding artery (orange arrow) could be barely depicted on pre-treatment imaging. **b** After DEB-TACE, this feeding artery became prominent (orange arrow) with residual tumor enhancement (red arrow) on posttreatment CTHA-EM image. A region of contrast stasis could be seen as a subtracted void area (white arrow). **c** Posttreatment native CT depicts contrast deposition from the delivery of DEB-TACE (white arrow) at the region suspicious for residual tumor enhancement. **d** Posttreatment CTHA identified a feeding artery (orange arrow) to the area of contrast deposition in **c**. However, it is not possible to distinguish residual enhancement from stasis without subtraction image. CT, Computed tomography; *CTHA*, CT hepatic arteriography; *CTHA-EM*, CTHA enhancement mapping; *DEB-TACE*, Drug-eluting beads transarterial chemoembolization

Among the 18 HCCs with residual arterial enhancement at CTHA-EM, a total of 22 feeding arteries were identified on the posttreatment CTHA-EM images. These feeding arteries were not depicted on posttreatment two-dimensional DSA and were obscured by the presence of contrast agent stasis and the enhancement of the liver parenchyma on posttreatment CTHA (Fig. 5). Of these 22 arteries, 20 (90.9%) were the same arteries initially identified on pre-treatment CTHA and treated with DEB-TACE (suboptimal embolization endpoint per CTHA-EM), whereas 2 (9.1%) arteries were not supplying the tumor on pre-treatment CTHA and were therefore designated as newly developed collateral arteries posttreatment.

## Discussion

In this proof-of-concept study, the proposed CTHA-EM method demonstrated a high accuracy in predicting HCC treatment response to DEB-TACE on the initial follow-up imaging. Moreover, among HCCs where complete response was not achieved, CTHA-EM was able to depict the feeding arteries supplying the residual tumor enhancement area. The deformable registration and subtraction of unenhanced and contrast-enhanced CTHA images allowed to differentiate true residual tumor enhancement from contrast agent stasis or pooling within the treated HCCs. Additionally, tumor relative enhancement metric provided an objective and reproducible method to determine residual tumor enhancement following DEB-TACE.

Objective response (complete or partial response) according to mRECIST is a relevant prognostic factor of survival in HCC patients undergoing TACE [30–32]. Furthermore, patients with complete response have longer overall survival than those with partial response [7]. Unfortunately, approximately only 50% of the treated HCC tumors exhibit complete response after DEB-TACE [30–32]. Although such low complete response rates can be attributed to tumor biology or more advanced disease [22, 33], technical limitations during TACE procedures cannot be neglected as potential contributing factors. An important technical limitation of DEB-TACE is the lack of an objective intraprocedural method to determine the treatment endpoint. Angiographic indicators of complete embolization are difficult to reproduce, resulting in variable survival outcomes [18]. Moreover, nondominant communicating arterial arcades, which frequently supply HCCs at the liver watershed areas, might become dominant feeding arteries when the primary feeding arteries are embolized [34, 35] or occluded [36, 37]. Such tumor perfusion redistribution from interlobar collateral arteries could be one of the reasons for the low complete response rates [38, 39]. Therefore, recognizing residual tumor enhancement and its feeding arteries intra-procedurally is crucial during DEB-TACE.

CTHA-EM can be a valuable intraprocedural tool to improve the treatment response prediction during HCC treatment with DEB-TACE. The challenges in predicting intra-procedural response to DEB-TACE are well illustrated in our present study, as 39.1% (18/46) of the treated HCCs had residual tumor on the first follow-up imaging, despite being deemed to have complete response at the DEB-TACE procedure’s completion per the interventional radiologist’s judgment based on the DSA and CTHA (non-contrast and arterial phase) images. This suggests that standard DSA and CTHA have a low negative predictive value in predicting incomplete DEB-TACE. Also, there is indication that CTHA-EM can provide valuable information on the identification of the residual tumor feeding artery, which could improve the treatment strategy (*i.e.,* need for further embolization), potentially improving the overall complete response rates following DEB-TACE.

We believe that the high accuracy of CTHA-EM in identifying residual tumor demonstrated in the present study is related to several factors. First, the use of CT has many advantages as it provides reliable and reproducible imaging, allows standardized quantitative arterial enhancement assessment via HU quantification, offers easy correlation with the follow-up imaging, and facilitates better image quality with comparable or often lower radiation exposure compared to CBCT [24–40]. Second, since CTHA-EM uses subtraction imaging, it removes background noise resulting from contrast-media stasis/pooling within the tumor and adjacent vessels and allows reliable HU value normalization. Based on the CT value comparison between non-residual and residual tumors, quantitative evaluation showed significant differences, suggesting that an incomplete embolization might have been the culprit for not achieving complete response on the vast majority of HCCs reported in this present patient population.

This study has several limitations. First, the small number of cases reported might limit the generalizability of the present findings. The use of CT during hepatic arteriography for DEB-TACE has been part of our institution’s practice since 2016. The requirement of pre- and posttreatment CTHA images limited the case number for this proof-of-concept study. Such repeated CTHA imaging is not performed routinely in all cases, and it would translate into an increase on overall patient exposure to radiation. Therefore, we included patients from three different institutions from 2017 to 2020 to expand the number of cases to achieve sufficient power. Likewise, there is an inherent sampling bias as only patients undergoing first session DEB-TACE and who did not have extrahepatic arterial supply were included in this study. Third, correlating the results of CTHA-EM to treatment response on first follow-up imaging has its limitations, as tumor progression or response may occur beyond the first follow-up imaging. Fourth, the applicability of this method is limited to procedure rooms equipped with hybrid angio/CT system. Although the install base of angio/CT equipment in interventional radiology practice has seen a recent uptick, its current availability is widely limited to major academic centers, thus presently resulting in limited availability of the proposed method when implemented for intra-procedural use.

In conclusion, the proposed CTHA-EM method can accurately and quantitively predict intraprocedural embolization endpoint and immediate treatment response after DEB-TACE on first imaging follow-up. In addition, it allows detection of the feeding arteries to residual tumor enhancement areas. The role of this method to personalize post-DEB-TACE imaging follow-up and its impact on tumor progression or response should be prospectively evaluated.

## Supplementary Information

**Additional file 1: Supplementary Table 1.** CTHA acquisition and reconstruction parameters.

## Data Availability

Anonymized clinical data specific to this study will be shared upon request.

## Abbreviations

CBCT: Cone-beam computed tomography
CT: Computed tomography
CTHA: Computed tomography hepatic arteriography
CTHA-EM: Computed tomography hepatic arteriography-enhancement mapping
DEB-TACE: Drug-eluting beads transarterial chemoembolization
DSA: Digital subtraction angiography
mRECIST: Modified response evaluation criteria in solid tumors
HU: Hounsfield units
MRI: Magnetic resonance imaging
ROI: Region of interest
TACE: Transarterial chemoembolization
