# Supplementary material for: A novel method for predicting hepatocellular carcinoma response to chemoembolization using an intraprocedural CT hepatic arteriography-based enhancement mapping: a proof-of-concept analysis
Source: Eur Radiol Exp. 2023 Jan 30;7:4. doi: 10.1186/s41747-022-00315-8 (PMC9886747; doi:10.1186/s41747-022-00315-8)
Supplement: Supplementary file 1 — Additional file 1: Supplementary Table 1. CTHA acquisition and reconstruction parameters. [file 41747_2022_315_MOESM1_ESM.pdf]

ELECTRONIC SUPPLEMENTARY MATERIAL

A novel method for predicting hepatocellular carcinoma response to chemoembolization using an intraprocedural CT hepatic arteriography-based enhancement mapping: a proof-of-concept analysis

Supplementary Table 1. CTHA acquisition and reconstruction parameters

	Institution A	Institution B	Institution C
Number of Patients	20	4	5
System	SOMATOM Definition Edge SG (Siemens Healthineers, Forchheim, Germany)	Aquilion ONE SG (Canon, Tokyo, Japan)	SOMATOM Definition AS SG (Siemens Healthineers, Forchheim, Germany)
Voltage	120 kV	120 kV	120 kV
mAs	300 reference mAs (CARE Dose)	noise index: 8	300 reference mAs (CARE Dose)
Rotation time	0.5 sec	0.5 sec	0.5 sec
Pitch	0.6	0.813	1
Collimated slices	64 x 0.6 mm	80 x 0.5 mm	32 x 0.6 mm
Reconstructed slice width	0.6 mm	0.5 mm	0.6 mm
Iterative reconstruction algorithm	Safire B40f	AiCE Body standard	Safire B40f
Reconstruction matrix	512*512	512*512	512*512
Field of view	40 cm	32 cm	30 cm
Quasi-isotropic voxel size	0.8 x 0.6 x 0.6 mm^3	0.6 x 0.6 x 0.5 mm^3	0.6 x 0.6 x 0.6 mm^3
Embolization guidance	Syngo embolization guidance (Siemens Healthineers, Forchheim, Germany)	Not used	Not used
Injection protocol			
Rate of injection (Varied depending on the DSA)	2mL/second, 8 seconds delay	2mL/second, 4 seconds delay	2mL/second, 8 seconds delay
Contrast dilution	Undiluted	75%	66.6%
Mean volume CT dose index ± SD (mGy) of proposed pre- and post-DEB-TACE dual-phase scans	86.2 ± 28.4	59.1 ± 10.4	40.0 ± 11.1
Mean volume CT dose index ± SD (mGy) of post-DEB-TACE dual-phase scans	43.2 ± 14.1	20.2 ± 5.7	29.4 ± 5.3

DSA digital subtraction angiography, *DEB-TACE* Drug-eluting beads transarterial chemoembolization; INR: International normalized ratio.
